# Change of the Smoking Behavior and Related Lifestyle Variables among Physicians in Fukuoka, Japan: A Longitudinal Study

**DOI:** 10.2188/jea.12.208

**Published:** 2007-11-30

**Authors:** Akihiko Kaetsu, Tetsuhito Fukushima, Masaki Moriyama, Takao Shigematsu

**Affiliations:** 1 Department of Hygiene, Faculty of Medicine, Tottori University.; 2 Department of Public Health, School of Medicine, Fukuoka University.; 3 Professor Emeritus, Fukuoka University.

**Keywords:** physician, prevalence of smoking, lifestyle, longitudinal study

## Abstract

Two cross-sectional surveys of the entire membership of the Fukuoka Prefecture Medical Association were conducted in 1983 and 1990 using a self-administered questionnaire, and respondents were registered as the study cohort. In this investigation the trend of the actual prevalence of smoking among physicians and the relationship between their smoking cessation and living habits were studied. A decline in the actual prevalence of smoking was demonstrated among the 2,656 subjects who responded to both surveys (2,543 men and 113 women). To examine any relationship between lifestyle variables and smoking cessation after 1983, those who were smokers in 1983 (1,099 men and 7 women, total 1,106) were divided into two groups, according to whether or not they still smoked in 1990. Subjects who quit smoking accounted for a larger proportion of those physicians with any of the following life-style variables: earlier bedtimes, unawareness of mental stress, greater consumption of fresh vegetables and fruit, and less coffee consumption. Further observation of the relationship between smoking cessation and changes in lifestyle variables showed that there were more who quit smoking among those who became employed physicians, had an increase of the body mass index (BMI) and began to be aware of mental stress. These results suggested that, for physicians who smoked, it is a possibility that smoking was an important way of coping with stress, and thus pointed to the need to take mental-health measures to help physicians to stop smoking.

## INTRODUCTION

The increasing mortality due to lung cancer and other smoking-related diseases in Japan^1^^)^ is a result of the high prevalence of smoking among Japanese adults – the highest of all advanced countries^2^^,^^3^^)^ – and planning effective measures for its reduction occupies an important place among Japan’s health policies^4^^)^. In order to reduce the prevalence of smoking in the nation, the reduction of the prevalence of smoking among physicians is considered to be a vital measure. This led to our examination of smoking status in a group of physicians.

In our previous report, it was suggested that physicians, as a group, could relatively easily stop smoking. This was also inferred from other reports that indicated notable declines in the prevalence of smoking and a higher proportion of ex-smokers among physicians than in the general population^5^^-^^10^^)^. Nevertheless, few studies followed up such physicians and demonstrated changes in their smoking status^11^^)^. Very little investigation of possible factors that might have been related to smoking cessation was conducted. Observation of what kinds of lifestyle variables might influence the success of later cessation of smoking was made by means of follow-ups of subjects in intervention programs for quitting smoking in the general population and in the workplace^12^^,^^13^^)^. Another report stated that differences in the success rate of smoking cessation appeared according to the psychological readiness of the smoker to quit^14^^)^. However, it is questionable whether or not those findings are directly applicable to smoking cessation programs for physicians, who constitute a group of a special nature. In addition, if one considers the report that the smoking habit is more difficult to modify or dislodge than habits of exercise or eating^15^^)^, it appears to be necessary to confirm that there have been alterations in daily habits that are regarded as predictors of smoking cessation. But very few reports have examined this^16^^)^. This study aimed to find any factors that might serve as predictors in a physician’s lifestyle of whether he or she would later quit smoking. For this purpose, the relationship between the lifestyle variables of physicians at the time of the baseline survey and the later status of their smoking habit were examined. Further, the alterations in the lifestyle variables themselves were verified over the seven-year follow-up period and their correlations with changes in smoking status were observed. Smoking cessation programs for physicians are discussed on the basis of the results.

## MATERIALS AND METHODS

Lifestyle surveys of the entire membership, as of both April 1, 1983 and April 1, 1990, of the Fukuoka Prefecture Medical Association were carried out in mid-1983 and mid-1990. The same self-administered questionnaire, which the subjects were requested to answer in their own names, was used in both surveys. The regional offices of the Medical Association carried out the distribution and collection of the questionnaires. In the baseline survey in 1983, 4,042 male physicians and 190 female physicians returned responses, totaling 4,232, for a gross return rate of 85%. In the follow-up survey in 1990, however, 3,565 (from 3,378 men and 187 women) were returned, giving a somewhat lower gross return rate of 63%. The number of subjects who responded in both surveys was 2,703 (2,584 men and 119 women), 64% of the baseline survey respondents (Table 1.). The details of survey method, items for lifestyle variables included in the questionnaire and the smoking status of the subjects were stated in our previous report.

**Table 1.** Age and sex distribution of respondents of both surveys.

Age	Sex		Total	( % )

	Male	Female		
-40 years	197	16	213	( 8.0 )
40-49 years	544	18	562	( 21.2 )
50-59 years	1,034	33	1,067	( 40.2 )
60-69 years	576	30	606	( 22.8 )
70+ years	192	16	208	( 7.8 )
Total	2,543	113	2,656	(100.0 )

Baseline				
Member	4,755	225	4,980	
respondents	4,042	190	4,232	( 85.0 )
Follow-up				
Member	5,323	341	5,664	
respondents	3,378	187	3,565	( 62.9 )

Age was classfied by the age at the baseline survey.

Of those who responded to both surveys, there were 2,656 physicians (2,543 men and 113 women) whose age, sex and smoking habits were known in both surveys. First, the McNemar’s test was performed to examine the significance of changes of smoking status in these subjects.

Next, for 1,106 subjects who were smokers at the time of the baseline survey (1,099 men and 7 women), the relations between the various lifestyle variables in the baseline survey – in particular, the possible predictive factors for physicians to quit smoking – and the smoking status at the time of the follow-up survey were examined. To determine statistical significance, the Mantel-Haenszel chi square test was used (p<0.05). Then, a univariate unconditional logistic regression analysis was done, in which each lifestyle factor was used as an independent variable, and abstention from smoking at the time of the follow-up survey as a dependent variable, and the odds ratio (OR) and the 95% confidence intervals (95%CI) were calculated. In addition, a step-wise multiple regression analysis with adjustment for age and sex was carried out on the items for which a statistically significant correlation was seen in a univariate unconditional logistic regression analysis.

Further, in relation to the various items examined in smokers at the time of the baseline survey, significance of changes that occurred between the two surveys were analyzed using the McNemar’s test. The SAS statistical software package, version 6.12 (SAS Institute, Caiy, NC) was employed for all statistical analysis.

## RESULTS

Age and sex distributions of respondents to both surveys who are the subjects of analysis in the present study are shown in Table 1. The proportion of subjects were 64% of the baseline survey respondents.

There was a significant decline in the actual prevalence of smoking in all age groups after 1983, as Table 2 indicates.

**Table 2.** The change of smoking prevalence by sex and age, Baseline(1983) and Follow-up(1990) survey.

Sex Age	N	1983		1990		Absolute Change (B-A)	Relative % Change (B-A)/A × 100	p
	
		Smoker	( % ) (A)	Smoker	( % ) (B)			
Male								
-40 years	197	106	53.8	84	42.6	-11.2	-20.8	**
40-49 years	544	222	40.8	176	32.4	-8.5	-20.7	***
50-59 years	1034	469	45.4	373	36.1	-9.3	-20.5	***
60-69 years	576	240	41.7	161	28.0	-13.7	-32.9	***
70+ years	192	62	32.3	40	20.8	-11.5	-35.5	***
Total	2543	1099	43.2	834	32.8	-10.4	-24.1	***

Female								
-40 years	16	0	0.0	0	0.0	0.0	-	-
40-49 years	18	1	5.6	0	0.0	-5.6	-100.0	-
50-59 years	33	3	9.1	3	9.1	0.0	0.0	-
60-69 years	30	2	6.7	2	6.7	0.0	0.0	-
70+ years	16	1	6.3	1	6.3	0.0	0.0	-
Total	113	7	6.2	6	5.3	-0.9	-14.3	-

Both combined								
-40 years	213	106	49.8	84	39.4	-10.3	-20.8	***
40-49 years	562	223	39.7	176	31.3	-8.4	-21.1	***
50-59 years	1067	472	44.2	376	35.2	-9.0	-20.3	***
60-69 years	606	242	39.9	163	26.9	-13.0	-32.6	***
70+ years	208	63	30.3	41	19.7	-10.6	-34.9	***
Total	2656	1106	41.6	840	31.6	-10.0	-24.1	***

Age was classfied by the age at the baseline survey

N: No. of subjects. Smoker: Current smoker

p; **: p<0.01, ***: p<0.001, statistically significant according to the McNemar test.

Of 1,106 smokers in the 1983 baseline survey, the proportion who had quit smoking by the time of the 1990 follow-up survey was analyzed for each of the 29 lifestyle variables examined in the previous report, and the results are presented in Table 3. The chi square test and univariate unconditional logistic regression analysis indicated significantly higher proportions of ex-smokers in relation to the following seven variables: age 60 years and over, the practice of internal medicine, earlier bedtime, no awareness of mental stress, regular physical activity, and daily consumption of fresh vegetables and of fruit. On the other hand, daily consumption of more than a cup of coffee was related to a significantly lower proportion of ex-smokers.

**Table 3.** The relationship between lifestyle variables and smoking cessation.

Variables	N	Quitters	(%)	p for chi-square	Relative risk	95%CI
1. Personal characters						
Sex						
Male	1,099	330	30.0		1.00	
Female	7	2	28.6	p= 0.933	1.07	0.21 - 5.55
Age						
-50 years	329	87	26.4		1.00	
50-59 years	472	124	26.3		1.01	0.73 - 1.39
60+ years	305	121	39.7	p= 0.001	0.55	0.39 - 0.77
Body Mass Index						
Under 22	410	116	28.3		1.00	
22 and over	696	216	31.0	p= 0.337	0.88	0.67 - 1.15
2. Physical conditions						
Subjective condition of health						
Not good	224	62	27.7		1.00	
Good	882	270	30.6	p= 0.393	0.87	0.63 - 1.20
Heart disease						
No	1,084	323	29.8		1.63	0.69 - 3.85
Yes	22	9	40.9	p= 0.261	1.00	
Peptic ulcer						
No	1,078	326	30.2		0.63	0.25 - 1.57
Yes	28	6	21.4	p= 0.315	1.00	
Health check-ups						
No	513	160	31.2		1.00	
Yes	593	172	29.0	p= 0.430	1.11	0.86 - 1.44
Customary medication						
No	503	153	30.4		0.97	0.75 - 1.25
Yes	603	179	29.7	p= 0.791	1.00	
3. Conditions of work						
Owner or employed						
Employed	182	46	25.3		1.00	
Owner	924	286	31.0	p= 0.127	0.76	0.53 - 1.08
Specialty of medical practice						
Surgery	410	105	25.6		1.00	
Internal medicine	696	227	32.6	p= 0.014	0.71	0.54 - 0.93
Number of working days						
Below 5	112	42	37.5		0.69	0.46 - 1.03
6 and over	994	290	29.2	p= 0.069	1.00	
Number of patients						
Less than 40	328	93	28.4		1.12	0.84 - 1.49
40 and over	778	239	30.7	p= 0.433	1.00	
Hours of consulting						
Less than 8	707	213	30.1		0.99	0.75 - 1.29
8 and over	399	119	29.8	p= 0.916	1.00	
4. Recreation						
Bedtime						
Before 12	709	236	33.3		0.64	0.48 - 0.84
After 12	397	96	24.2	p= 0.002	1.00	
Duration of sleep						
-6 or 8+	242	65	26.9		1.00	
6 to 8	864	267	30.9	p= 0.225	0.82	0.60 - 1.13
Physical fatigue						
Aware	898	268	29.8		1.00	
Unaware	208	64	30.8	p= 0.793	0.96	0.69 - 1.33
Mental stress						
Aware	807	224	27.8		1.00	
Unaware	299	108	36.1	p= 0.007	0.68	0.51 - 0.90
5. Exercise						
Current habit of exercise						
No	554	151	27.3		1.00	
Yes	552	181	32.8	p= 0.045	0.77	0.59 - 0.99
6. Food intake						
Miso soup (Misoshiru)						
Less than daily	585	174	29.7		1.00	
Daily	521	158	30.3	p= 0.833	0.97	0.75 - 1.26
Japanese pickles (Tsukemono)						
Less than daily	481	141	29.3		1.00	
Daily	625	191	30.6	p= 0.654	0.94	0.73 - 1.22
Bread						
Less than daily	716	209	29.2		1.00	
Daily	390	123	31.5	p= 0.416	0.90	0.69 - 1.17
Milk						
Less than daily	707	199	28.1		1.00	
Daily	399	133	33.3	p= 0.071	0.78	0.60 - 1.02
Fresh vegetables						
Less than daily	469	119	25.4		1.00	
Daily	637	213	33.4	p= 0.004	0.68	0.52 - 0.88
Yellow and green vegetables						
Less than daily	746	213	28.6		1.00	
Daily	360	119	33.1	p= 0.126	0.81	0.62 - 1.06
Fruit						
Less than daily	623	163	26.2		1.00	
Daily	483	169	35.0	p= 0.002	0.66	0.51 - 0.85
7. Beverage						
Coffee						
Less than daily	705	234	33.2		1.00	
Daily	401	98	24.4	p= 0.002	1.54	1.17 - 2.03
Green tea						
Less than daily	133	36	27.1		1.00	
Daily	973	296	30.4	p= 0.429	0.85	0.57 - 1.27
Alcohol - frequency						
Less than daily	496	140	28.2		1.00	
Daily	610	192	31.5	p= 0.241	0.86	0.66 - 1.11
Alcohol - amount per day						
Under 1 go	532	159	29.9		1.00	
1 go and over	574	173	30.1	p= 0.927	0.99	0.76 - 1.28

p for chi-square was based on Mantel-Haenszel chi-square test.

Relative risk and 95% CI were based on univariate unconditional logistic regression analysis.

Seven lifestyle variables for which significant OR were seen with univariate unconditional logistic regression analysis were used as independent variables, and age and sex were employed as adjustment factors in step-wise multiple logistic regression analysis. Table 4 presents the following five variables showing a statistically significant relationship with quitting smoking: earlier bedtime, no awareness of mental stress, daily consumption of fresh vegetables, fruit, and daily consumption of one or more cups of coffee.

**Table 4.** Multiple logistic regression analysis for smoking cessation.

Variables	Relative risk	95%CI
Bedtime		
Before 12	0.72	0.54 - 0.97
After 12	1.00	
Mental stress		
Aware	1.00	
Unaware	0.71	0.53 - 0.94
Fresh vegetables		
Not daily	1.00	
Daily	0.75	0.56 - 0.98
Fruit		
Not daily	1.00	
Daily	0.69	0.52 - 0.91
Coffee		
Not daily	1.00	
Daily	1.49	1.12 - 1.99

Relative risk and 95% CI were based on a step-wise multiple logistic regression analysis after adjusted for sex and age.

**Table 5.** The change of the lifestyle variables among smokers at baseline (1983) survey.

Variables	Quitters			Continue smokers		
	
	Baseline	Second	p	Baseline	Second	p
Body Mass Index						
22 and over	65.1%	73.5%	***	62.0%	64.5%	n.s.
Subjective condition of health						
Good	81.3%	60.2%	***	79.1%	73.8%	**
Heart disease						
No	97.3%	88.6%	***	98.3%	96.0%	**
Health check-ups						
Yes	51.8%	68.4%	***	54.4%	59.6%	**
Customary medication						
No	46.1%	59.6%	***	45.2%	53.0%	***
Owner or employed						
Owner	86.1%	79.5%	***	82.4%	85.5%	**
Number of working days						
Below 5	12.7%	23.8%	***	9.0%	14.2%	***
Number of patients						
Less than 40	28.0%	41.3%	***	30.4%	38.2%	***
Hours of consulting						
Less than 8	64.2%	78.6%	***	63.8%	76.1%	***
Bedtime						
Before 12	71.1%	78.9%	***	61.1%	65.2%	**
Duration of sleep						
6 to 8	80.4%	73.2%	*	77.1%	71.4%	**
Mental stress						
Unaware	32.5%	26.2%	*	24.7%	25.6%	n.s.
Yellow and green vegetables						
Daily	35.8%	50.6%	***	31.1%	35.7%	*
Alcohol - frequency						
Daily	57.8%	50.9%	**	54.0%	50.4%	*
Alcohol - amount per day						
1 go and over	52.1%	46.4%	*	51.8%	49.0%	n.s.

n.s.; not significant, *;p<0.05,**;p<0.01,***;p<0.001

statistically significant according to the McNemar test.

To investigate the relationship of changes of lifestyle variables and smoking status, the smokers at the time of the baseline survey (1983) were divided into two groups, quitters and continuing smokers, according to their smoking status at the follow-up survey (1990). Changes of lifestyle variables in the period between the two surveys were observed and the rates of changes were, in general, larger in quitters than in continuing smokers. The lifestyle variables that showed significant changes among those who quit smoking after the baseline survey are listed in 5. Those with BMIs of 22 and over, owner-practitioners, and those with no awareness of mental stress showed the marked differences between the quitters and continuous smokers. The relationships between the changes in these variables and the discontinuation of smoking are given in Table 6. Increases in BMI to more than 22, change in conditions of work, and awareness of mental stress showed a significant correlation in multiple regression analysis.

**Table 6.** The relationship between the change of lifestyle variables, which showed different trend between quitters and continue smokers, and smoking cessation.

	N	Quitter	Quit rate	(%)	OR	95%CI
Body Mass Index						
Decrease, Stable	986	286	29.0		1.00	
Increase	120	46	38.3	p= 0.035	0.66	0.44 - 0.98

Owner or employed						
Stabel, to be Owner	1061	304	28.7		1.00	
to be Employed	45	28	62.2	p= 0.001	0.31	0.17 - 0.59

Mental stress						
Stable, to be unaware	963	276	28.7		1.00	
to be aware	143	56	39.2	p= 0.011	0.65	0.45 - 0.94

P for chi-square was based on Mantel-Haenszel chi-square test. Odds ratios and 95% CI were based on unconditional logistic regression analysis after adjusted for sex and age.

## DISCUSSION

### Effect of gender in the present study

The present study analyzed the relationships of changes in lifestyle and in smoking status to the 8-year follow-up data for the physician cohort. It is considered necessary to take the sex gender of the subjects into account in the investigation of the habit of smoking^17^^)^. However, this study used the entire body of data as a whole (men and women together) because its main concern was in the changes of lifestyle and smoking status among the subjects, and there were very few women physicians (0.1%, 7 of 1,106 smokers cohort) in the study cohort. Gender did not appear to have any effect on the study results.

### What can be expected in the follow-up study

The previous study reported the relationship between lifestyle variables and current abstinence from smoking on the basis of the results of a cross-sectional survey of a group of physicians. In the present study, the relationships between lifestyle variables at the time of the baseline survey (1983) and smoking status at the follow-up survey (1990) were examined. Those subjects who were smokers in the baseline survey were divided according to their smoking status at the time of the follow-up survey into two groups, quitters and continuing smokers, and the lifestyle variables among quitters were compared with those among continuing smokers. In such an analysis, it was hoped that it would be possible to clarify what kind of relationships existed between lifestyle variables and changes in smoking habits.

### Changes in the prevalence of smoking

The proportion of current smokers in each age group at the baseline survey and that in the follow-up survey were compared and significant decreases were observed in every age group. While the relative decline in the prevalence of smoking among physicians under 60 years of age was about 20%, that in the over-60s was more than 30%. An investigation on smoking among elderly people found that many of them quit smoking after the age of 60^18^^)^. A similar tendency was observed among physicians.

### Predictors of smoking cessation- When these variables are present, more physicians quit smoking.

In our previous, cross-sectional study, owner-practitioners showed a higher prevalence of smoking than employed physicians. In the present study, although the difference was not statistically significant, more owner-practitioners than employed physicians tended to discontinue smoking (OR=0.76, 95%CI: 0.53-1.08). There is a report that a person’s smoking habit is affected by the smoking status of those in his surroundings, such as family members, co-workers and others^12^^)^. Many owner-practitioners in Japan are less affected in this way because there are, in general, few co-workers in their clinics with such close contact, and such a work environment may be of assistance in the process of smoking cessation^19^^)^.

In the present study, a statistically significant relationship was found between a change in smoking behavior and the practice of internal medicine. This suggests that the working conditions in internal medicine are a factor in the discontinuation of smoking among physicians. One of the reasons for the discontinuation of smoking by a physician which is suggested in the literature, although with not such high priority, is related to the sense of professional responsibility that he or she ought to set an example of a healthy lifestyle for patients^7^^,^^10^^)^. Sixty-two percent of the smoking physicians in the present study were internists who had the opportunities to consider such aspects of smoking. Although this factor was not selected as a statistically significant variable by multiple regression analysis after sex and age were used as adjustment factors, it is thought to be important and deserving of consideration in relation to smoking by physicians.

The statistically significant relationships between changes of smoking habit and bedtime, awareness of mental stress, consumption of fresh vegetables, fruit and coffee were seen in both univariate regression analysis and multiple regression analysis after adjustment for sex and age. Since most clinics and hospitals in Japan commence consultations about 9 a.m., the physician’s bedtime needs to be before midnight in order to ensure the 7-8 hours of daily sleep that was proposed by Breslow^20^^)^. However, although duration of sleep showed no statistically significant relationship, a higher proportion of those who gave up smoking (OR=0.82, 95%CI: 0.60-1.13) were seen in the physicians who slept for 6-8 hours per day. Maintenance of regular sleep of suitable duration (one aspect of a healthy lifestyle) is thought to have an effect on quitting smoking.

In relation to mental stress, a significantly higher proportion of those who quit smoking was observed among physicians with unaware of mental stress than among those who were aware of stress. It has been demonstrated that continuing smoking or relapsing after discontinuing is affected by stress^21^^,^^22^^)^. Also, in a study on smoking among nurses, it was reported that the more nurses smoke, the more they are stressed^23^^)^. Since physicians tend to experience stress as long as they continue to practice^24^^)^, it might prevent them from quitting smoking. However, most of the physicians surveyed (1,073 subjects, 97%) continue practicing, and their reasons for quitting smoking appeared to be that they were suffering neither from a disease nor from a declining physical condition, unlike the case of elderly people in the general population^18^^)^. Regarding the change of drinking habits, 113 of the 610 smoking physicians who drank alcohol every day gave up daily drinking, but the proportion who quit drinking was less than 20%. This may suggest that smoking, which is more deleterious to the health than drinking, was abandoned purposefully and positively among physicians.

In the behavioral sciences, it is recommended to develop a habit of physical exercise as one method of overcoming the habit of smoking^25^^)^. The results of univariate regression analysis suggested that this was an effective approach because smoking physicians who have the habit of taking regular exercise tended to quit smoking. However, physical exercise was not selected by multiple regression analysis after adjustments for sex and age as a statistically significant variable. If physical activity is considered as a method of coping with stress, a correlation between awareness of mental stress and exercise habits could be expected. In the present study, out of 807 physicians who are aware of being under stress, 392 physicians take regular exercise. On the other hand, 160 of 299 physicians who were not aware of having such stress also have the habit of exercising. In the chi square test, the relationship between mental stress and exercise as a habit was not significant (p=0.145). Considering the effects of physical activity together with the results of our cross-sectional study, exercise did not appear to act as an effective method of stress coping, since there were few physicians who both smoked and took regular exercise.

As for the changes in lifestyle variables, among those who quit smoking, there was a significant decrease in the proportion of physicians who unaware of mental stress, while among persistent smokers, this proportion showed no notable change, but just a slight increase. These findings suggested that discontinuance of smoking might bring an awareness of stress to the surface if smoking plays any role, consciously or not, as a means of coping with stress for smoking physicians.

The results suggest that relieving mental stress is effective in reducing the prevalence of smoking among physicians. It is hoped that any measure effective for stress coping for the benefit of the mental health of physicians will be developed so that the prevalence of smoking among physicians would decrease and result in a reduction in the prevalence of smoking among the local population as well as in the mortality due to smoking-related diseases.

## CONCLUSIONS

The present study led to the following conclusions:

1. Physicians who consume more fresh vegetables and fruits, limit their coffee drinking, and maintain a proper sleeping schedule-that is, more health-conscious in their own daily lives, smoke less or find it easier to stop smoking.

2. Mental stress was thought to be one of the causes of persistent smoking, and it is important from the standpoint of occupational health that methods be devised for terminating the habit.

3. It was realized that owner-practitioners showed lower prevalence of smoking and higher rate of smoking cessation. Physicians, as health professionals, are relatively health conscious and have a full recognition of the hazards of smoking. Owner-practitioners, especially, ought to be models of healthy lifestyle, both individually and as a group, for the local population, and so their smoking habits may have a major influence on the smoking of those around them.
